# Susceptibility of *Armigeres subalbatus* Coquillett (Diptera: Culicidae) to Zika virus through oral and urine infection

**DOI:** 10.1371/journal.pntd.0008450

**Published:** 2020-07-06

**Authors:** Chun-xiao Li, Xiao-xia Guo, Yong-qiang Deng, Qin-mei Liu, Dan Xing, Ai-juan Sun, Qun Wu, Yan-de Dong, Ying-mei Zhang, Heng-duan Zhang, Wu-chun Cao, Cheng-feng Qin, Tong-yan Zhao

**Affiliations:** 1 Department of Vector Biology and Control, State Key Laboratory of Pathogen and Biosecurity, Institute of Microbiology and Epidemiology, Beijing, China; 2 Department of Virology, State Key Laboratory of Pathogen and Biosecurity, Institute of Microbiology and Epidemiology, Beijing, China; 3 Department of Epidemiology, State Key Laboratory of Pathogen and Biosecurity, Institute of Microbiology and Epidemiology, Beijing, China; INDEPENDENT RESEARCHER, UNITED STATES

## Abstract

**Background:**

Zika virus (ZIKV) disease outbreaks have been occurring in South America since 2015, and has spread to North America. Because birth defects and cases of Guillain Barré have been associated with infection with ZIKV, this has drawn global attention. ZIKV is generally considered an *Aedes-*transmitted pathogen. The transmission of ZIKV through blood by *Aedes* mosquito bites has been recognized as the major transmission route. However, it is not clear whether there are other transmission routes that can cause viral infection in mosquitos. The aim of the present study is to describe the susceptibility of *Armigeres subalbatus*, which often develop in human waste lagoons, to ZIKV, through oral infection in adult mosquitoes and urine infection in larvae.

**Methodology/Principal findings:**

Five-day-old female *Ar*. *subalbatus* ingested infectious blood meals containing ZIKV. After 4, 7, and 10 days of ingesting infectious blood meals, ZIKV could be detected in the midguts, salivary glands, ovaries, and collected saliva of mosquitoes. The ZIKV infection rate (IR) on day 10 reached 40% in salivary glands and 13% in saliva, indicating that these mosquitoes were able to transmit ZIKV. In addition, ZIKV infection was also discovered in mosquito ovaries, suggesting the possibility of vertical transmission of virus. Moreover, *Ar*. *subalbatus* transmitted ZIKV to infant mice bitten by infectious mosquitoes. In a second experiment, 1^st^-instar larvae of *Ar*. *subalbatus* were reared in water containing ZIKV and human urine. After pupation, pupae were placed in clean water and transferred to a mosquito cage for emergence. Although ZIKV RNA was detected in all of the larvae tested, ZIKV was not detected in the saliva of any adult *Ar*. *subalbatus*. Considering that there are more uncontrollable factors in nature than in the laboratory environment, the possibility that the virus is transmitted to adult mosquitoes via larvae is very small period.

**Conclusions/Significance:**

Adult *Ar*. *subalbatus* could be infected with ZIKV and transmit ZIKV through mosquito bites. Therefore, in many rural areas in China and in undeveloped areas of other Asian countries, the management of human waste lagoons in the prevention and control of Zika disease should be considered. Corresponding adjustments and modifications should also be made in prevention and control strategies against ZIKV.

## Author summary

Zika is a serious threat to global health since the outbreak in Brazil in 2015. Zika virus (ZIKV) is generally considered as an *Aedes-*transmitted pathogen. The transmission of ZIKV by mosquito bite has been recognized as the major transmission route. However, it is possible that some important potential vectors may not yet have been examined. *Armigeres subalbatus* is one of primary mosquito species in China, which could develop in human waste lagoons. In the study, through oral infection experiments, it was found that *Ar*. *subalbatus* could be infected with ZIKV and transmit ZIKV through mosquito bites. Therefore, in many rural areas in China and in undeveloped areas of other Asian countries, it is valid to consider that this mosquito species should be given enough attention in the prevention and control of Zika epidemic.

## Introduction

Zika virus (ZIKV) is a mosquito-borne virus that can cause disease in humans. Its outbreak during the end of 2015 and the first half of 2016 in many countries in South America, such as Brazil, Colombia, Puerto Rico, and Venezuela, caused more than a million Zika cases [1]. In addition, more than one thousand cases of microcephaly in infants have also been confirmed to be associated with ZIKV infection in pregnant women to date [2].

ZIKV has been detected in *Aedes* mosquitos, including *Ae*. *africanus*, *Ae*. *luteocephalus*, *Ae*. *Aegypti*, and *Ae*. *albopictus* from Senegal, Ivory Coast, Burkina Faso, Central African Republic, Uganda, and locations in Asia [3–5]. *Ae*. *aegypti*, *Ae*. *albopictus* and *Ae*. *hensilli* are known to be potential vectors of ZIKV through experimental infection and transmission studies [6–10]. Although ZIKV is generally considered an *Aedes-*transmitted pathogen [11], it is a member of the *Spondweni serocomplex* that is close to the Japanese encephalitis virus (JEV) serocomplex according to phylogenetic analysis and E protein structure [12–16]. Both JEV and ZIKV are in the family Flaviviridae. The question remains whether mosquitoes that transmit JEV can also be infected with and transmit ZIKV.

It is important to know that which mosquito species are susceptible to infection and can transmit ZIKV In order to preventand control of Zika disease. Current studies generally agree that oral infection and transmission of ZIKV through mosquitoes ingesting blood from patients. However, it is not known whether mosquitoes can be infected by ZIKV through other routes. Recently, some studies have confirmed that ZIKV can survive in human urine for some time [17–18]. Thus, is it possible that mosquito larvae reared in water contaminated with ZIKV might become infected and adults derived from these larvae could transmit ZIKV?

*Armigeres subalbatus* Coquillett is a common mosquito species in the integrated regions between rural and urban areas in Asia and usually develop in human waste lagoons [19]. Its density is usually high, and this mosquito species represents a major pest species in China [20]. Some field surveys and laboratory assessments showed that *Ar*. *subalbatus* is one of the major transmission vector of JEV [21–24]. In addition, some arboviruses, such as Getah virus, Banna virus, and Kadipiro virus, have been isolated from *Ar*. *subalbatus* in the field [25–27]. A recent investigation found that ZIKV could be isolated from the field-collected *Ar*. *subalbatus* in Guizhou Province of China [28]. Due to its susceptibility to flaviviruses and its breeding characteristics, *Ar*. *subalbatus* is a potential transmission vector of ZIKV in China.

To assess the susceptibility of *Ar*. *subalbatus* to ZIKV, infection experiments were performed in this study using two methods, oral infection via blood meal in adult mosquitoes and rearing of larvae in urine water containing ZIKV to test the possibility of urine infection of ZIKV in mosquitoes.

## Materials and methods

### Ethics statement

All experimental protocols involving animals were approved by the Laboratory Animal Center, State Key Laboratory of Pathogen and Biosecurity, Beijing Institute of Microbiology and Epidemiology IACUC (Institutional Animal Care and Use Committee) (BIME 2011–09). The study of animals was conducted in strict accordance with the recommendations in the Guide for the Care and Use of Laboratory Animals of the National Institutes of Health.

### Mosquitoes

For the experimental infection, *Ar*. *subalbatus* was collected from Hainan Province of southern China in the summer of 2012 and reared in the laboratory. The mosquitoes were maintained under standard insectary conditions at 26±1°C and 75±5% relative humidity (RH), with a photoperiod of 14 h:10 h light: dark (L:D) cycles. Prior to the infectious feed, adult mosquitoes were provided with 10% sugar water.

### Virus

The contemporary ZIKV strain SZ01 (GenBank Nos. KU866423) was used to expose the mosquitoes as previously described in Deng et al., 2016 [29]. The stock virus used in the current study was passaged in C6/36 cells prior to the infectious feed.

### Oral infection experiment: Oral infection of female mosquitoes with ZIKV

Five-day-old female mosquitoes were starved for 16 hours prior to the infectious blood meal. The blood meal consisted of 1:1 mouse blood and virus suspension. Oral infections with ZIKV were performed with a virus titer (VT) of 6.5 log_10_ PFU/ml, verified by titration in a standard plaque assay. Mosquitoes were fed with an infectious blood meal warmed to 37°C using a Hemotek membrane feeding system housed in a feeding chamber. After 30 minutes of blood feeding, mosquitoes were cold-anesthetized, and fully engorged females were transferred to 300-mL plastic cups, maintained at 29±1°C and 75±5% RH with a 14 h/10 h L:D cycle, and provided with 10% sugar water.

### Oral infection experiment: Mosquito processing

To determine the ZIKV infection and dissemination rates in *Ar*. *subalbatus* females, 15–25 female mosquitoes were sampled at 4, 7, and 10 days post exposure (dpe). To prevent cross-contamination of virus between the midgut, salivary glands, and ovary of each mosquito, these organs were carefully dissected using fresh dissecting needles, and the organs were iteratively rinsed in phosphate-buffered saline (PBS) three times each. The midgut, salivary glands, and ovaries from each mosquito were individually transferred to 1.5-mL microcentrifuge tubes containing 100 μL of Dulbecco’s modified Eagle’s medium (DMEM) (GIBCO^TM^, Invitrogen, Beijing, China) supplemented with 2% fetal bovine serum (FBS). These organs were homogenized using 5-mm stainless steel grinding balls (Next Advance, Averill Park, NY, USA) in a Bullet Blender^TM^ 24 mixer mill (Next Advance) set at a frequency of 12/sec for 1 min. All dissecting needles were dipped in 80% ethanol, burned, and cleaned before re-use.

### Oral infection experiment: Transmission analysis

To determine the ZIKV transmission rates of mosquitoes, saliva of *Ar*. *subalbatus* was collected from individual female mosquitoes at 4, 7, and 10 dpe as previously described in Guo et al., 2016 [11]. Briefly, the wings and legs of each mosquito were removed, and the proboscis was inserted into a quartz capillary containing 10 μL of FBS. After 45 min, FBS containing saliva was expelled in 100 μL of DMEM medium for detection.

To determine the ability of ZIKV transmission by *Ar*. *subalbatus*, 10 1-day-old infant mice were put into the cage containing ~100 mosquitoes which had been fed on a viremic blood meal 8 days previously. After the mosquitoes had fed for 2 hours, the infant mice were removed and transferred to an isolation facility for biosafety. All the infant mice were euthanized and the brain of each individual mouse was dissected on days 7–10 after the mice had been fed upon by the mosquitoes. A weight of 100mg brain tissue was individually transferred to 1.5 mL microtubes containing 100 mL of DMEM (GIBCO^TM^, Invitrogen, Beijing, China) supplemented with 2% FBS.

### Urine infection experiment: Urine-virus mixture preparation

A total of 450 mL of clean water was prepared in a white porcelain plate, two kinds of urine-virus mixture were poured into water: (1) The virus is added to the water together with the urine; (2) The virus suspension is first mixed with the urine, and after 30 minutes, the urine-virus mixture is added to the water together. After mixing for 1h, 12h and 24h, both a plaque assay and a nucleic acid detection were used to detect whether there is Zika virus in the mixture solution. To determine if it was even possible for larvae reared in ZIKV-contaminated water to become infected, we chose to use a high virus titer for subsequent experiments.

### Urine infection experiment: Mosquito larvae rearing

A total of 450 mL of clean water was poured into a white porcelain plate, and 50 mL of healthy adult (26-year-old female) urine was added and mixed thoroughly. Hatched 1^st^-instar larvae of *Ar*. *subalbatus* were placed in the mixture solution. During the whole larval stage (1^st^-4^th^ instar) of mosquitoes, 1 mL of virus solution was added into the urine solution every day (day 0 to 11). After pupation, pupae were placed in clean water and transferred to a clean mosquito cage for emergence. Each day (d 3–12), 1 mL of the ZIKV-urine-water suspension was collected and frozen at -80°C for subsequent virus detection.

### Urine infection experiment: Mosquito processing

When larvae grew to the 4^th^ instar in the urine solution, 10 larvae were collected, and each one was washed by clean water in the petri dish for three times and removed to a 1.5-mL microcentrifuge tube containing 100 μL of DMEM supplemented with 2% FBS and then frozen in -80°C for subsequent virus detection.

Five days after mosquitoes emerged into adult mosquitoes, saliva was collected from 60 female mosquitoes, and their salivary glands, midguts, and ovaries were dissected and transferred to 1.5-mL microcentrifuge tubes containing 100 μL of DMEM supplemented with 2% FBS. In addition, 20 male mosquitoes were collected and placed in 1.5-mL microcentrifuge tubes with one individual in each tube. All samples were frozen at -80°C for subsequent virus detection.

### Detection of virus

Total RNA was isolated from the midguts, salivary glands, ovaries and saliva of the female mosquitoes, the whole body of the male mosquito and the ZIKV-urine-water suspension samples, using a QIAamp Viral RNA Mini Kit (Qiagen, Germany) following the manufacturer’s recommendations. ZIKV in these organs was detected using a One-Step Detection Kit for Zika Virus RNA (Daan Gene, Guangzhou, China), and PCR was conducted as previously described Li et al., 2017 [29].

The number of viral RNA copies was calculated by generating a standard curve from RNA isolated from uninfected mosquitoes by titrating with a known amount of seed virus, the titer of which was determined using a plaque assay [29]. The amount of RNA is expressed as log_10_ RNA copies/mL. The standard RNA used in the nucleic amplification assays was extracted from virus dilutions of a known titer as determined by a plaque assay.

To confirm the presence of infection virus in the mosquitoes, the experiment was repeated and a subset of the samples was tested by the plaque assay. The virus titers of midguts and salivary glands collected from 30 females were detected through plaque assay at 10 dpe. The homogenates of midguts and salivary glands were serially diluted and inoculated onto monolayers of BHK cells in 12-well plates. Cells were incubated for 7 days at 37°C and were then stained with a crystal violet solution (0.2% in 10% formaldehyde and 20% ethanol). Titers were expressed in plaque-forming units (pfu).

### Infection and transmission analysis

The infection rate (IR) of the midguts, salivary glands and ovaries on each sampling day was calculated by dividing the number of infected midguts, salivary glands and ovaries by the total number of mosquitoes tested. The transmission rate (TR) was calculated by dividing the number of mosquitoes with infected saliva by the total number of mosquitoes tested.

### Statistical analysis

All statistical tests were conducted using SPSS 13.0 software (IBM, USA). Rates were compared using Fisher’s exact test. Kruskal-Wallis tests were used to determine differences in the viral titers for the two mosquito strains at different times. P-values > 0.05 were considered non-significant.

## Results

### Susceptibility of *Ar*. *subalbatus* to ZIKV through oral infection

The results showed that viruses could be detected in the midguts, salivary glands, and ovaries of *Ar*. *subalbatus* after 4, 7, and 10 days of ingesting infectious blood meals containing ZIKV (Table 1). The IR of midguts was not significantly different (Fisher’s exact test, P > 0.205) at 4, 7, and 10 dpe, with 43% (25/60) of all of the mosquitoes being infected. However, the IR of the salivary glands was higher at day 10 than at either day 4 or 7 dpe, but the difference was not significant (Fisher’s exact test, P > 0.440). ZIKV could also be detected in the collected saliva, albeit with a slightly lower IR than that in salivary glands. The IR in the ovaries was relatively lower than that in the midguts and salivary glands, however, the ovarian IR also reached 16% (4/25) at 10 dpe.

**Table 1:** ZIKV infection rate and quantification of Zika viral RNA copies, by real-time RT-PCR, detected in the salivary gland, midgut, ovary and collected saliva of *Ar*. *subalbatus* females at 4, 7, and 10 dpe (blood meal).

Dpe	Rate	Midgut	Salivary gland	Ovary	Saliva
4	IR^a^	53% (8/15)	13% (2/15)	7% (1/15)	7% (1/15)
	VT^b^	5.7±1.3	5.1±3.3	7.4	2.7
	TR^c^	7% (1/15)			
7	IR^a^	32% (8/25)	16% (4/25)	12% (3/25)	12% (3/25)
	VT^b^	6.4±1.6	6.1±1.9	6.1±2.2	5.4±1.0
	TR^c^	12% (3/25)			
10	IR^a^	36% (9/25)	28% (7/25)	16% (4/25)	8% (2/25)
	VT^b^	6.7±2.1	4.7±2.1	5.7±2.5	4.0±0.9
	TR^c^	8% (2/25)			

^a^ IR (infection rate) of the midguts, salivary glands, ovaries and larvae on each sampling day was calculated by dividing the number of infected midguts, salivary glands, ovaries and larvae by the total number of mosquitoes tested.

^b^ VT (virus titer) is expressed as log_10_ RNA copies/mL (± the standard deviation).

^c^ TR (transmission rate) is calculated by dividing the number of infected saliva samples by the total number of mosquitoes tested.

After *Ar*. *subalbatus* mosquitoes ingested infectious blood meals with ZIKV, the VT in the midguts, salivary glands and ovaries of mosquitoes had significant individual differences. The VT in some individuals reached more than 8.0 log_10_ RNA copies/mL, while some individuals only had 2.0 log_10_ RNA copies/mL. Therefore, the mean value had larger variations among different days. A subset of the samples were tested for the presence of infection virus, and titers of virus were consistent with those determined by qRT-PCR. The virus titers of midguts and salivary glands collected from 30 female adults were 3.6 and 1.4 log_10_ pfu/mL on day 10 post-feeding on the viremic blood meal, respectively.

ZIKV RNA was detected in the mosquito saliva, which indicated that this virus could be transmitted by mosquitoes. The presence of ZIKV was detected in the saliva of mosquitoes at 4, 7, and 10 dpe. The VT reached the value of 2.7, 5.4±1.0, and 4.0±0.9 log_10_ RNA copies/mL at 4, 7, and 10 dpe, respectively.

More than one red blotch was present on the skin of all 10 infant mice after removal from the virus-infected mosquito cage, which indicated all of the infant mice were bitten by at least one mosquito. Nearly all, 9/10 (90%) of the infant mice had viral RNA in their brain after being bitten by infectious mosquitoes, with an average estimated virus titre of 6.9±1.7 log_10_ RNA copies/mL. Thus, the detection of ZIKV RNA in the mice bitten by the ZIKV-exposed mosquitoes is direct evidence that *Ar*. *subalbatus* can transmit ZIKV.

### Susceptibility of *Ar*. *subalbatus* to ZIKV through urine infection

Two different ways of making the urine-virus-water suspension were evaluated. When the virus and urine were added together into water, the viral RNA copy number after 1h, 12h, and 24h were 6.1±0.1, 5.8±0.1, and 4.5±0.2 log_10_ RNA copies/mL, respectively. When tested for infectious by plaque assay, the ZIKV titers at 1h and 12h were 2.2±0.8 and 1.9±1.2 log_10_ pfu/mL, respectively. However, no plaques were detected after 24h. When the virus was first mixed with urine, and the urine-virus mixture added to the water 30 minutes later, the viral RNA copy numbers after 1h, 12h, and 24h was 5.6±0.1, 5.0±0.2, and 4.9±0.1 log_10_ RNA copies/mL, respectively. When these samples were tested for infectious by plaque assay, ZIKV was only detected at 1h, with 2.7±1.7 log_10_ pfu/mL. No plaques were detected after 12h or 24h.

**Fig 1:**
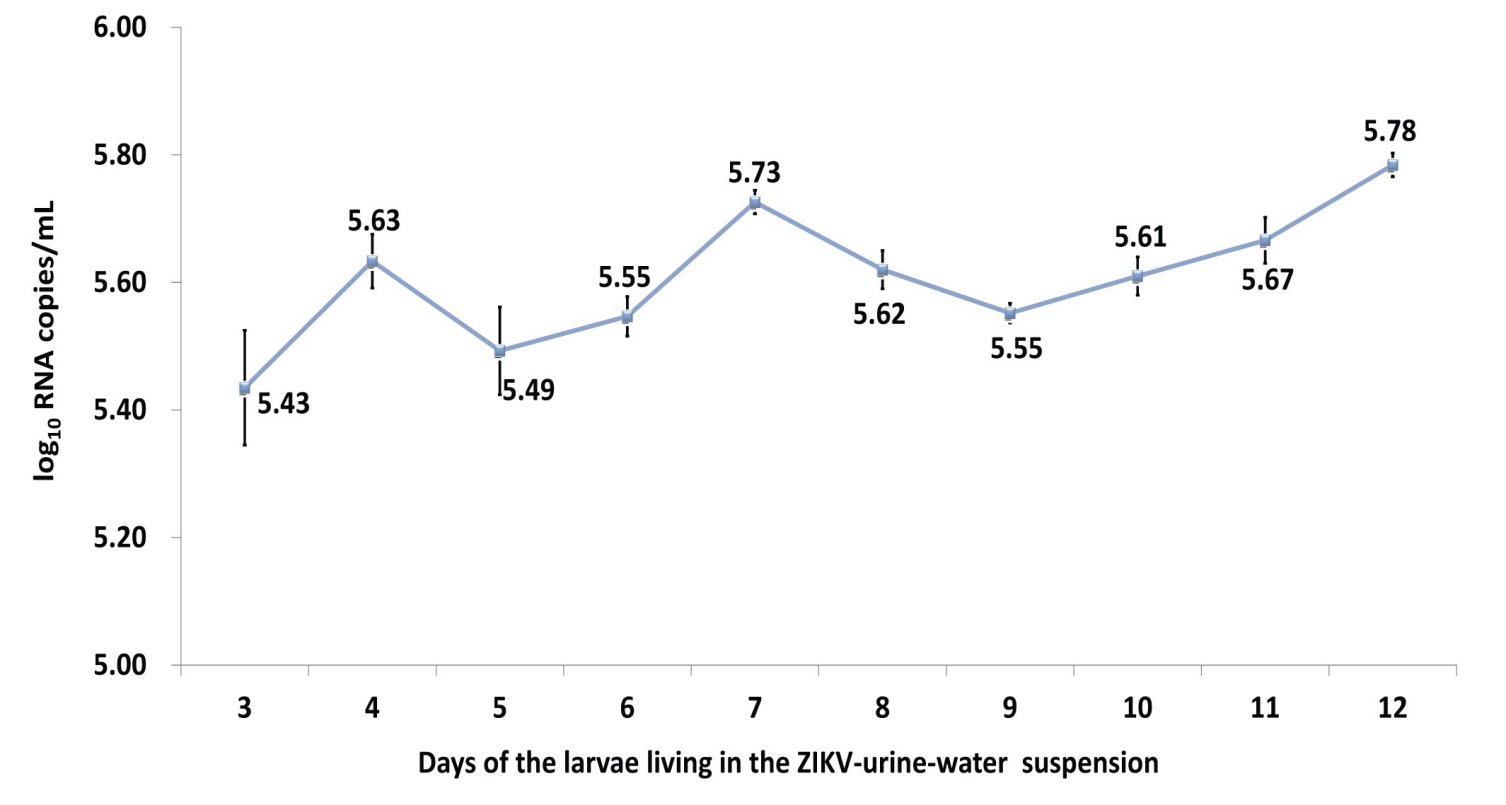
Quantification of Zika viral RNA copies detected in the ZIKV-urine-water suspension that larvae were reared. (There was no significant difference in the viral RNA titers in the continuous sampling on day 3–12; however, no infectious virus was detected on any of the sampling days).

As the purpose of the study was to determine the posibility of larvae becoming infected if they were reared in water to which ZIKV urine had been added, the experimental design was made to make it more likely for this to occur (i.e., ZIKV added to diluted urine) to see if this was possible. If it did not occur under these conditions, then it would be extremely unlikely to occur in the more natural one where the virus was in the pure urine before being added to the water. Therefore, based on the above results, the first mixture method was selected with a longer virial survival time for the next experiment. *Ar*. *subalbatus* larvae were reared in a urine and water mixture containing ZIKV. While the titer of ZIKV RNA remained fairly constant from days 3–12 (Fig 1), no infectious virus was detected from any of these samples despite fresh virus being added daily.

Although ZIKV RNA was detected in 100% of the 4th instar larvae and that the VT reached 4.0 log_10_ RNA copies/mL, the detected RNA found in larvae that might be residual virus in midgut that had been swallowed by larvae together with food, or be residual nucleic acid on the surface of the larvae. After the mosquitoes emerged as adults, the quantitative PCR results were all negative. Similarly, viruses could not be detected from the saliva of any adult.

## Discussion

*Armigeres subalbatus* is a competent vector of ZIKV. This is important because *Ar*. *subalbatus* is a major mosquito pest in China, which often develop in human waste lagoons [19, 20]. Experiments of oral infection in adult mosquitoes was conducted, the results showed that *Ar*. *subalbatus* could be infected with ZIKV, similar to other mosquitoes with confirmed ability to be infected with and transmit ZIKV. In addition, the results in the collected saliva showed that *Ar*. *subalbatus* had the ability to transmit ZIKV. However, its ability to transmit ZIKV infection was lower than that of *Ae*. *aegypti* and *Ae*. *albopictus* [30–32]. In the transmission experiment, infant mice were infected after being bitten by virus-positive mosquitoes and disseminated to and replicated in the mouse brains. The findings verified that *Ar*. *subalbatus* could be a potential vector to transmit ZIKV. Moreover, ZIKV RNA were detected in the ovaries, suggesting that *Ar*. *subalbatus* may also transmit ZIKV transovarially, similar to *Ae*. *aegypti* and *Ae*. *albopictus* [29, 31, 32].

In the urine infection experiment, although the larvae were immersed in water with high-titered ZIKV RNA and RNA was detected in 100% of the larvae, the lack of infected adults indicated that the ZIKV detected at the larval stage may represent viruses in water that were swallowed together with food by the larvae. Most viruses could not break the midgut infection barrier of the larvae and would only be digested. Although the results showed there was no significant difference in RNA titers in the continuous sampling from day 3 to 12, indicating that the ZIKV RNA remained stable in the environment where the larvae grew, no plaques were detected after 24 hours of its introduction, indicating that the virus titer was less than 1.0 log_10_ pfu/mL. Therefore, infectious ZIKV did not appear to be stable in a water-urine suspension. The mere detection of RNA does not always mean that virus is present. Even though the larvae were exposed to actual infectious virus for up to 24 hours, none of them appeared to have become infected. Thus, given the extremely low titers of infectious ZIKV in human urine [17], it is unlikely for mosquitoes to become infected via this route in nature.

In this study, we demonstrated that *Ar*. *subalbatus* was a competent vector of ZIKV because it became infected and was able to transmit virus after ingestion of a virus-infected bloodmeal. In a recent report, ZIKV was detected and isolated from the field-collected *Ar*. *subalbatus*, suggesting the important role of this mosquito species in the circulation of arboviruses [28]. Although the mere isolation of virus from a species does not prove it could be a vector in the real world. However, our study proved that *Ar*. *subalbatus* is able to transmit ZIKV after ingesting virus. This discovery presents new challenges for the global prevention and control of ZIKV. *Ar*. *subalbatus* is a common species of mosquito and widely distribute in many rural areas in China and in undeveloped areas of other Asian countries, the role of *Ar*. *subalbatus* as a vector should be considered. The management of human waste lagoons should be included in the prevention and control of Zika disease.
